# Red-Shift Effect and Sensitive Responsivity of MoS_2_/ZnO Flexible Photodetectors

**DOI:** 10.1186/s11671-015-1151-5

**Published:** 2015-11-16

**Authors:** Yu-Jen Hsiao, Te-Hua Fang, Liang-Wen Ji, Bo-Yi Yang

**Affiliations:** National Nano Device Laboratories, National Applied Research Laboratories, Tainan, 741 Taiwan; Department of Mechanical Engineering, National Kaohsiung University of Applied Sciences, Kaohsiung, 807 Taiwan; Institute of Electro-Optical and Materials Science, National Formosa University, Yunlin, 632 Taiwan

**Keywords:** MoS_2_, Photodetectors, Photo-induced response, Flexible

## Abstract

The optoelectronic characteristics of molybdenum disulfide (MoS_2_)/ZnO flexible photodetectors are investigated. A red-shift effect and improved photocurrent properties of the flexible devices are demonstrated. MoS_2_ doping improved the photocurrent properties and conductivity. The photocurrent/dark current ratios of pure ZnO and MoS_2_/ZnO flexible photodetectors were 10^3^ and 10^4^, respectively. The responsivity of MoS2/ZnO increased, and the wavelength was red-shifted.

## Background

Molybdenum disulfide (MoS_2_) is a promising candidate for optoelectronic sensors because of its unique semiconducting channel when used as a phototransistor [1]. MoS_2_ phototransistors have recently been integrated with conventional semiconductor circuitry [2]. Bulk MoS_2_ is an indirect-gap semiconductor with a bandgap of 1.2 eV [3], whereas a single-layer MoS_2_ is a direct-gap semiconductor with a bandgap of 1.8 eV [4]. The photodetector (PD) has a broad spectral range, with photocurrent that monotonously increases as the wavelength of incident light is decreased from 680 to 400 nm. Two-dimensional and single-layer ultrasensitive MoS_2_ PDs have a photoresponsivity that is 10^6^ better than that of the first graphene PDs (~0.5 mA W^−1^) [5]. In addition, a high-performance complementary inverter and selective gas sensing based on MoS_2_ field-effect transistors was studied [6–8].

There are various ways of synthesizing MoS_2_ nanostructures including electrochemical/chemical synthesis [9], laser ablation [10], solution-based exfoliation [11], and chemical vapor deposition (CVD) [12]. Another method, the rapid vibro-milling technique, was employed for investigating the potentiality of obtaining nano-sized powders. MoS_2_ nanoparticles obtained using vibro-milling, which can be applied at an industrial scale, have good solubility and biocompatibility. However, few applications of MoS_2_ synthesized by rapid vibro-milling have been reported. The rapid vibro-milling process was employed for investigating its potential for obtaining nanometer-sized powders [13]. The electro-optical properties of ZnO compounds have been studied extensively [14]. To our knowledge, MoS_2_/ZnO films have not been thoroughly investigated, which is the motivation for this research. The morphology and photoresponsivity properties of the MoS_2_ nanocrystals on ZnO film are studied and discussed.

## Methods

Nanocrystalline MoS_2_ (from Alfa Aesar, 325 mesh, 99 %) was prepared using a high-energy ball-milling method. MoS_2_ was milled in ceramic milling vials (zirconia) using zirconia balls for 10, 20, and 40 h. The ball-to-powder weight ratio was 2:1 to produce at least 2 g of nanopowder. The mechanical milling was performed in a horizontal oscillatory mill (Retsch, PM 400) operating at 25 Hz. The as-synthesized materials were characterized by X-ray diffraction (XRD, Rigaku Dmax-33). The morphology and microstructure were examined using atomic force microscopy (AFM, Bruker) and transmission electron microscopy (TEM, Hitachi HF-2000).

**Fig. 1:**
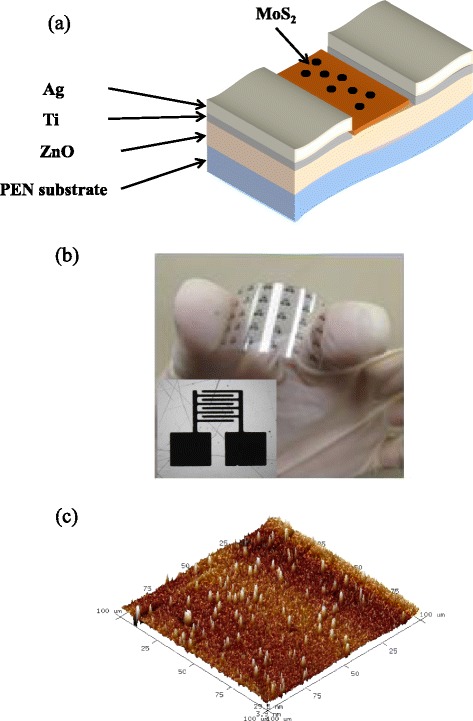
**a** Schematic diagram of flexible MoS_2_/ZnO PD. **b** Optical image of MoS_2_/ZnO PD fabricated on PEN substrate. *Inset* shows electrode pattern structure. **c** AFM image of 5 wt% MoS_2_ nanocrystal coating on ZnO/PEN substrate

In this work, MoS_2_/ZnO was used to fabricate metal-semiconductor-metal (MSM) ultraviolet (UV) PDs, as shown in Fig. 1a. ZnO thin films were deposited on polyethylene naphthalate (PEN) substrates using radio frequency (RF) magnetron sputtering. During growth, the working pressure of the chamber was about 5 × 10^−2^ Torr, the RF power was 250 W, and the gas mixing ratio (Ar/O_2_) was 10:1. The thickness of the ZnO film was 100 nm. Ag/Ti electrodes were used to provide ohmic contact on the ZnO film. They were deposited using the electron beam evaporation method. The fingers of the Ag contact electrodes had a width of 20 μm, a length of 250 μm, and a space of 20 μm. In 10 cm^3^ of alcohol, 0, 0.1, and 0.2 g of MoS_2_ nanopowder were dissolved, respectively. The PD materials of MoS_2_ were spin-coated with a rotation speed of 300 RPM in the air. The photocurrent, dark current, and responsivity of the PDs were measured using an HP4156C semiconductor parameter analyzer. The spectral response of the PDs was measured with a light source which employed a 300-W xenon lamp as the light source and a monochromator covering the range of 300–700 nm.

## Results and Discussion

Figure 1b shows an optical image of the flexible PDs with MoS_2_ coated on the ZnO/PEN substrate. The PDs exhibited a transmission of above 80 % and high bending strength. The bending curvature radius was larger than 10 mm. The Ag electrode pattern is shown in the inset of Fig. 1b. The interdigital electrodes have eight fingers with a fixed length of 2000 μm and a width of 50 μm. The spin-coated 5 wt% MoS_2_ nanocrystals on the ZnO/PEN substrate were also characterized using AFM to better understand the morphological properties with a large area of 100 × 100 μm, as shown in Fig. 1c. The pure ZnO film has a root-mean-square (rms) roughness of 13.2 nm, and the spin-coated MoS_2_ on the ZnO fim has that of 84.9 nm. In this study, the particle size of MoS_2_ was around 20~50 nm. During the spin-coating of MoS_2_, the nanocrystals had a uniform morphology and monodispersity. They were deposited on the ZnO/PEN substrate due to the gravitational force, causing the high roughness on the ZnO/PEN substrate.

**Fig. 2:**
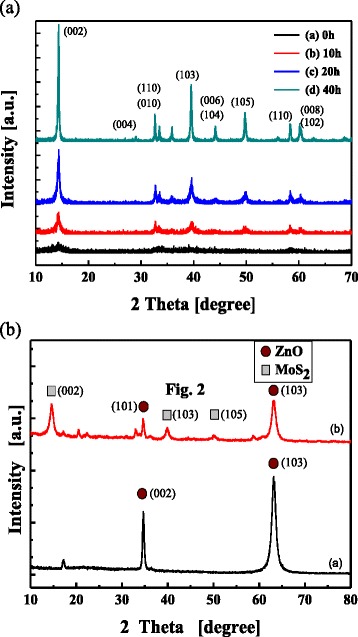
**a** XRD patterns of MoS_2_ obtained with various high-energy ball-milling durations, showing peak broadening due to reduced crystallite size. **b** XRD pattern of ZnO film and ZnO coated with nanocrystal MoS_2_

MoS_2_ exhibits the characteristic peaks of a polycrystal structure in the XRD analysis, as shown in Fig. 2a. The XRD patterns also have a strong (002) peak, with a preferred orientation at 2*θ* = 10.47. No secondary phases were detected for the pure MoS_2_ samples. Nanocrystalline MoS_2_ was obtained using high-energy ball-milling. The full width at half maximum of the diffraction peak is rather small, which indicates that the film crystallinity is fairly good for the pristine MoS_2_ powder. The average grain size was determined by the line broadening of XRD patterns for various milling times. Many hours of milling were sufficient to produce nanocrystalline powders.

The average grain sizes were determined from XRD according to Scherrer’s equation [15]:

1 $$ D = \frac{k\lambda }{\beta \cos \theta } $$

where *D* is the average grain size, *k* is a constant (equal to 0.89), *λ* is the X-ray wavelength (equal to 0.1542 nm), *θ* is the (002) peak angle, and *β* is half the peak width. The average grain size of powders milled for 20 h was 28.4 nm. The line broadening of the nanocrystalline samples is due to the small grain size and strain-induced response [16]. Figure 2b shows the XRD patterns of ZnO and ZnO films with 5 wt% MoS_2_ (~28 nm) coating layer. A (002) peak at 34.5° along with a strong (103) peak was observed for the ZnO thin films, indicating the polycrystalline nature of the thin films. The peak position of ZnO remains almost unchanged because the bottom ZnO layers were controlled to have a thickness of 100 nm. Therefore, the nanocrystalline MoS_2_ can be observed on the ZnO thin film by grazing incidence XRD.

**Fig. 3:**
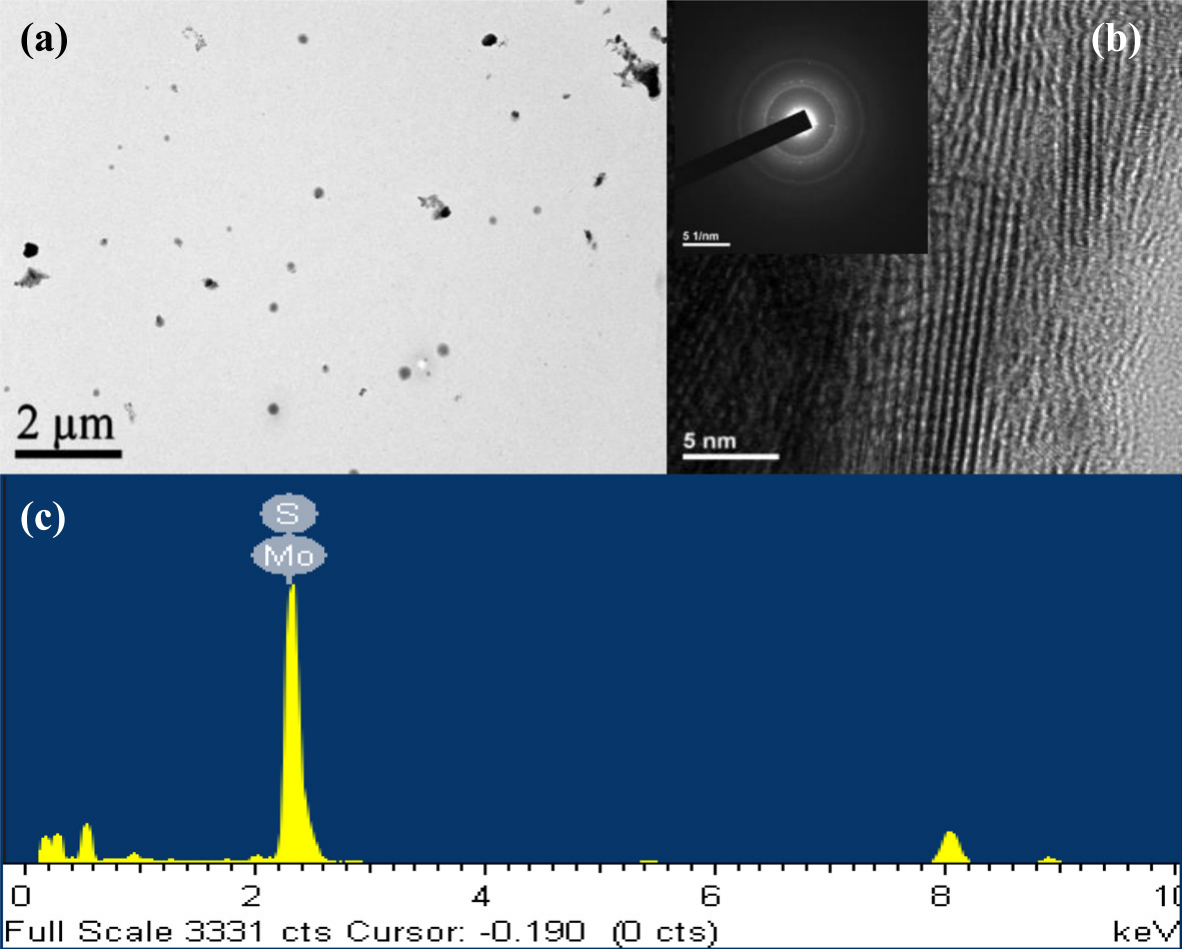
**a** TEM images of as-synthesized MoS_2_ nanocrystals obtained with high-energy ball-milling for 40 h, **b** high-resolution TEM image and electron diffraction pattern of nanocrystal, and **c** EDS analysis of MoS_2_ nanocrystal

Figure 3a shows a low-magnification TEM image. Spherical nanocrystals can be clearly observed. The diameter of the nanocrystals was about 20–50 nm. Figure 3b shows a high-resolution TEM image, indicating the periodic atom arrangement of the MoS_2_ nanosheets at a selected location. The interplanar distances of the crystal fringes are about 5.08 Å, corresponding to the spacing d-[100] of hexagonal MoS_2_ (JCPDS card no. 77-1716). A polycrystalline phase was present in the MoS_2_ matrix. The well-defined selected area electron diffraction (SAED) pattern clearly shows the diffraction spots in the inset of Fig. 3b. The energy-dispersive X-ray spectroscopy (EDS) line profiles indicate that the nanocrystal consists of Mo and S as shown in Fig. 3c. The signals were generated by the nanobeam incident to the nanocrystal MoS_2_.

**Fig. 4:**
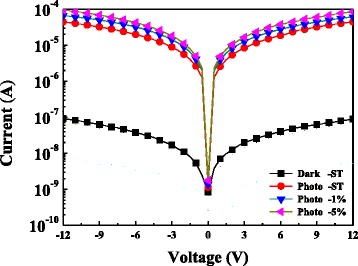
Room-temperature *I*-*V* characteristics of PD coated with MoS_2_ layer at various concentrations measured in the dark and under 390-nm UV illumination

Figure 4 shows the current-voltage (*I*-*V*) characteristics of the PDs with different MoS_2_ layers under dark and illumination conditions. The measurements were conducted at 5-V bias and 340-nm illumination. The results show that the light current of the photodetectors with MoS_2_ layers was enhanced. The highest photocurrent was obtained for the device with a 5 wt% MoS_2_ layer. The photocurrent to dark current contrast ratios of the 0, 1, and 5 wt% MoS_2_ PDs biased at 5 V were 8840, 13,100, and 17,800, respectively. MoS_2_ thus increased optical absorption. The dark current of the MoS_2_ film was very small. This may be due to the low background carrier concentration. The photo-generated holes recombined with the surface-adsorbed oxygen ions. The results show that hole and electron pairs were generated when UV light illuminated the ZnO layer. PD devices based on MoS_2_ thus exhibit a very high photoresponsivity [1, 17]. The high performance can be ascribed to the straight electron transport path offered by nanocrystalline MoS_2_ powders. The MoS_2_ nanopowders have a high surface-to-volume ratio. A heterojunction forms at the interface between ZnO and MoS_2_ nanopowders.

**Fig. 5:**
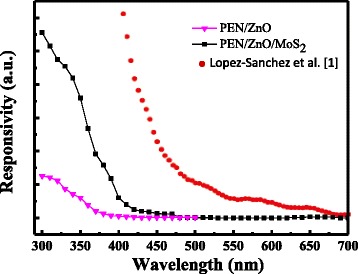
Photoresponsivity of ZnO, ZnO/MoS_2_, and monolayer MoS_2_ device as a function of illumination wavelength

Figure 5 shows the photoresponsivity of pure ZnO and 5 wt% MoS_2_/ZnO PDs in the UV-to-visible light region. The device with MoS_2_ nanopowders shows a red shift (from 360 to 420 nm) and increased photocurrent. The photoresponsivity of the composite MoS_2_/ZnO device as a function of illumination wavelength was measured. The response increased as the illumination wavelength was reduced from 420 to 300 nm. The higher responsivity of the 5 wt% MoS_2_/ZnO PD compared with that of ZnO is attributed to the improved carrier transport and collection efficiency [18]. The adsorbates on the MoS_2_ surface or at the MoS_2_/ZnO interface affect not only the carrier transport behaviors but also the photoelectrical responses [19]. The optical properties of nanocrystalline MoS_2_ have been measured by UV/VIS absorption spectroscopy technique. The optical absorption spectrum of MoS_2_ nanocrystallines shows a minimum optical absorption feature of about 400 nm and strong rising absorption edge shifts towards the UV region. Therefore, the prepared MoS_2_/ZnO MSM photodetectors generated photoresponse between 300 and 420 nm UV range and diminishing photoresponse at the visible range. A good agreement is observed between the MoS_2_ nanocrystalline absorption characteristics and the photoresponsivity data. Lopez-Sanchez et al. demonstrated ultrasensitive monolayer MoS_2_ phototransistors with improved device mobility and ON current [1]. A thin film of MoS_2_ nanocrystals has also been demonstrated through laser ablation that it could be used as a material for the fabrication of UV PDs [17]. This flexible MoS_2_/ZnO optoelectronic devices could be used in fields of low-light imaging sensors.

## Conclusions

MoS_2_ nanopowder was deposited on flexible devices using high-energy ball-milling method. Flexible ZnO and MoS_2_/ZnO MSM PDs were investigated. The results show that the photocurrent/dark current ratios of pure ZnO and MoS_2_/ZnO flexible PDs were 10^3^ and 10^4^, respectively. The responsivity increased and the wavelength was red-shifted when a 5 wt% MoS_2_ layer was used. There was a significant improvement in the photo-induced properties.
